# Morphometric integration and modularity in configurations of landmarks: tools for evaluating a priori hypotheses

**DOI:** 10.1111/j.1525-142X.2009.00347.x

**Published:** 2009-07

**Authors:** Christian Peter Klingenberg

**Affiliations:** Faculty of Life Sciences, The University of Manchester, Michael Smith BuildingOxford Road, Manchester M13 9PT, UK

## Abstract

Identifying the modular components of a configuration of landmarks is an important task of morphometric analyses in evolutionary developmental biology. Modules are integrated internally by many interactions among their component parts, but are linked to one another only by few or weak interactions. Accordingly, traits within modules are tightly correlated with each other, but relatively independent of traits in other modules. Hypotheses concerning the boundaries of modules in a landmark configuration can therefore be tested by comparing the strength of covariation among alternative partitions of the configuration into subsets of landmarks. If a subdivision coincides with the true boundaries between modules, the correlations among subsets should be minimal. This article introduces Escoufier's *RV* coefficient and the multi-set *RV* coefficient as measures of the correlation between two or more subsets of landmarks. These measures can be compared between alternative partitions of the configuration into subsets. Because developmental interactions are tissue bound, it is sensible to require that modules should be spatially contiguous. I propose a criterion for spatial contiguity for sets of landmarks using an adjacency graph. The new methods are demonstrated with data on shape of the wing in *Drosophila melanogaster* and the mandible of the house mouse.

## INTRODUCTION

Organisms are integrated to function as a whole, but this integration is not uniform throughout (e.g., Olson and Miller 1958). Individuals and their major morphological units are composed of multiple parts that are more or less distinct of each other due to function, anatomical structure, and embryological origins. This coordination into subunits has long been known as morphological integration (Olson and Miller 1958; Cheverud 1996;) and has become the focus of renewed interest in evolutionary developmental biology under the heading of modularity (Raff 1996; Wagner 1996; von Dassow and Munro 1999; Bolker 2000; Winther 2001; Schlosser and Wagner 2004; Callebaut and Rasskin-Gutman 2005; Klingenberg 2008;). Integration and modularity concern the degree of covariation between parts of a structure, which can be studied by means of morphometric methods. An important task for morphometric research is to determine whether a structure is a single integrated unit or consists of several distinct modules, and if so, to identify the modules. Integration and modularity have been investigated in many different study systems such as insect wings (Birdsall et al. 2000; Klingenberg and Zaklan 2000; Zimmerman et al. 2000; Klingenberg et al. 2001;), rodent mandibles (Atchley and Hall 1991; Atchley et al. 1992; Cheverud et al. 1997; Ehrich et al. 2003; Klingenberg et al. 2003, 2004; Monteiro et al. 2005; Márquez 2008; Zelditch et al. 2008), and the skulls of various mammals, including humans (Cheverud 1982, 1995; Leamy et al. 1999; Lieberman et al. 2002; Bookstein et al. 2003; Ackermann 2005; Bastir and Rosas 2005; Goswami 2006a; Mitteroecker and Bookstein 2008).

**Fig. 1:**
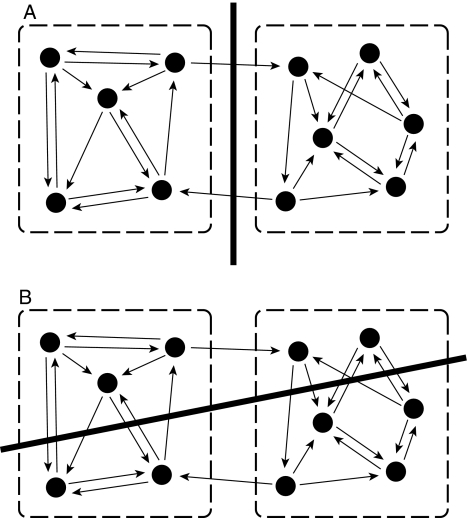
Delimiting modules by comparing different partitions of the structure. Each diagram shows two modules (dashed lines) whose parts are integrated internally by many interactions (arrows), but which are relatively independent of each other because there are only few interactions between modules. The bold lines indicate two ways to partition the overall structure into two subsets. (A) The subdivision coincides with the boundary between modules. (B) The subdivision does not coincide with the modular boundary and therefore goes across both modules. Note that the dividing line in (B) intersects many more arrows than in (A). Accordingly, there are more interactions between the parts in the two subsets, and a stronger covariation between subsets is expected.

A primary task for morphometric studies of modularity is to delimit modules and to evaluate hypotheses about their boundaries. A module is a unit whose parts are integrated tightly because there are many and often strong interactions among them, but different modules are relatively independent of each other because the interactions between modules are fewer or weaker (e.g., Klingenberg 2008). Therefore, inferences about the boundaries of modules from the patterns of covariation among traits can be made by partitioning the traits into subsets in different ways and comparing the degree of covariation between subsets (Fig. 1; Klingenberg 2008). If the division of the traits into subsets coincides with the boundary between modules, the covariation between the subsets results from the few or weak interactions between traits belonging to different modules (arrows across the bold line in Fig. 1A). Accordingly, the degree of correlation between the subsets should be relatively low. If the division into subsets is not congruent with the boundary between modules, however, it cuts across the modules and some of the covariation between the subsets is from the numerous and strong interactions within modules (many arrows cross the bold line in Fig. 1B). Accordingly, the covariation between the traits in the two subsets is expected to be stronger. Such comparisons of different ways to divide a set of traits into subsets provide a method to test hypotheses about the boundaries of modules. A division into subsets that correspond to the true modules should result in a smaller degree of covariation among modules than other ways of partitioning the traits into subsets. Alternatively, if the covariation between subsets of traits that correspond to hypothesized modules is just as strong or stronger as a large proportion of the alternative partitions, the hypothesis of modularity can be rejected because a central prediction is not met.

This article introduces methods that implement this approach to assess hypotheses about modularity in the context of geometric morphometrics (Bookstein 1996; Dryden and Mardia 1998;). Geometric morphometrics has opened up new possibilities for the study of morphological integration, but poses specific challenges for the study of integration and modularity. This article introduces the *RV* coefficient of Escoufier (1973) as a scalar measure of the strength of association between the coordinates of two sets of landmarks and presents a new generalization of this measure for multiple sets of landmarks. Hypotheses about the boundaries between modules can be evaluated by partitioning the configuration in different ways and comparing the *RV* coefficients between subsets of landmarks. For contexts where the interactions that define modules take place within continuous tissues, I provide a method for limiting the comparisons specifically to subsets of landmarks that are spatially contiguous. Finally, I briefly discuss the effect of allometry and similar phenomena that might enhance integration across modules. Some of these methods improve on or replace the methods used in previous analyses of integration in the *Drosophila* wing and the mouse mandible (Klingenberg and Zaklan 2000; Klingenberg et al. 2003, 2004). In this article, I use both fly wings and mouse mandibles as examples to demonstrate the new methods.

## EXAMPLE DATA

**Fig. 2:**
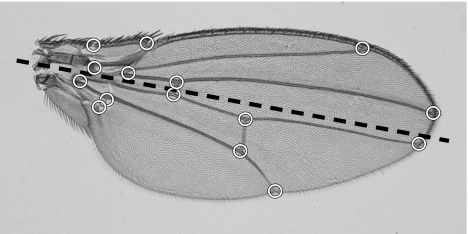
Wing of *Drosophila melanogaster* with the landmarks used in the example (circles) and the approximate location of the boundary between the anterior and posterior developmental compartments (dashed line).

To illustrate the methods discussed throughout this article, I use two data sets concerning individual variation and fluctuating asymmetry in fly wings and in mouse mandibles. The flies were a sample of 109 female *Drosophila melanogaster* (Oregon-R strain) reared under standard laboratory conditions. For each fly, digital images of the left and right wings were taken and a set of 15 landmarks was digitized (Fig. 2). A preliminary analysis of measurement error using Procrustes ANOVA (Klingenberg and McIntyre 1998) showed that its effect on shape amounted to <5% of the component of fluctuating asymmetry, and was therefore negligible. A previous study, with a slightly smaller set of landmarks, found that the entire wing is a single integrated module (Klingenberg and Zaklan 2000). Here I use the new methods to reassess this result and to test it against the alternative hypothesis that the anterior and posterior compartments (Fig. 2) are distinct modules (e.g., Thompson and Woodruff 1982; Cavicchi et al. 1991; Pezzoli et al. 1997;).

**Fig. 3:**
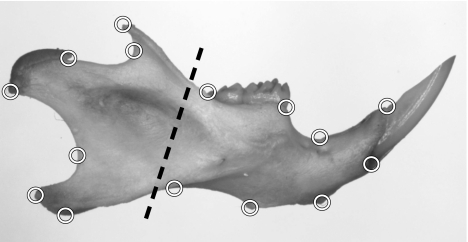
A mouse mandible with the landmarks used in the analysis (circles). The dashed line indicates the boundary between the alveolar region (to the right) and the ascending ramus (to the left), which have been suggested as possible modules.

A contrasting second data set of mouse mandibles is used to illustrate the method for locating the boundary between modules. Several studies have investigated the subdivision into two main modules, the alveolar region in the anterior part of the mandible and the ascending ramus in the posterior part (Atchley et al. 1985; Cheverud et al. 1991, 1997; Leamy 1993; Mezey et al. 2000; Ehrich et al. 2003; Klingenberg et al. 2003, 2004). A set of 15 landmarks (Fig. 3) was digitized on the mandibles of 90 mice for both the left and right sides. Details on the specimens, the landmarks, the procedures for correcting the effects of size and group structure, as well as a full analysis of this data set have been published elsewhere (Klingenberg et al. 2003).

For both data sets, the landmark configurations from both sides were included in a generalized Procrustes fit (with appropriate reflections; Klingenberg and McIntyre 1998; Klingenberg et al. 2002;). The averages of the configurations of each individual were used to compute the among-individual covariance matrices, and the coordinate differences of left and right sides (averaged over the replicate measurements) were used to compute the covariance matrices of fluctuating asymmetry (for details of the methods, see Klingenberg and McIntyre 1998; Klingenberg et al. 2002;). These covariance matrices are the basis for the further analyses described below.

## QUANTIFYING AND TESTING COVARIATION WITHIN A LANDMARK CONFIGURATION

Because the strength of covariation between different regions of a structure is the criterion for assessing integration and modularity in morphometric data (Fig. 1), a measure for quantifying covariation between sets of landmarks is of critical importance. I recommend the *RV* coefficient (Escoufier 1973) as measure of association to replace the use of the trace correlation (Hooper 1959) used recently in a similar context (Klingenberg et al. 2003, 2004). The trace correlation shows undesirable statistical behavior, for example, in models where the entire variation is contained in a subspace of shape space (e.g., a pure allometric model). Moreover, it also suggests spuriously high covariation between sets if the sample size is small (Mitteroecker and Bookstein 2007, Fig. 3).

### The *RV* coefficient

This section introduces Escoufier's (1973)*RV* coefficient as a scalar measure of the strength of the association between two sets of variables (see also Robert and Escoufier 1976; Robert et al. 1985; Cléroux and Ducharme 1989;). The two sets of variables are contained in the random vectors **x**_1_ and **x**_2_, consisting of *p* and *q* variables, and can be written as a combined random vector **x**=(**x**_1_, **x**_2_) of length *p*+*q*. This combined vector of variables defines a covariance matrix that is patterned as follows: 
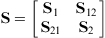


The diagonal blocks **S**_1_ and **S**_2_ correspond to the covariance matrices of the two sets of variables each on its own, whereas the off-diagonal block **S**_12_ is the matrix of covariances between the variables of the two sets (the matrix **S**_21_ is the transpose of **S**_12_).

The *RV* coefficient is calculated as follows: 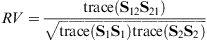


The trace of a square matrix is the sum of its diagonal elements. This formula can be interpreted as an extension of the expression for the squared correlation coefficient between two variables. The term trace(**S**_12_**S**_21_) in the numerator is the sum of the squared covariances between the two sets of variables. This has been used as a measure of the total amount of covariation between two sets of variables in the context of partial least squares analysis (Bookstein 1991, p. 43; Rohlf and Corti 2000). Similarly, the terms trace(**S**_1_**S**_1_) and trace(**S**_2_**S**_2_) in the denominator can be interpreted as measures of the total amounts of variation in the two sets of variables. The entire expression therefore represents the amount of covariation scaled by the amounts of variation within the two sets of variables, which is analogous to the calculation of the correlation coefficient between two variables. Note, however, that the *RV* coefficient uses squared measures of variances and covariances, and is therefore more directly comparable to a squared correlation coefficient.

The *RV* coefficient takes values between zero and one, and has a number of useful mathematical properties (Escoufier 1973; Robert and Escoufier 1976; Cléroux and Ducharme 1989;). It is zero if all covariances between the two sets of variables are zero (all elements of **S**_12_), which means that the two blocks of variables are completely uncorrelated with each other. The *RV* coefficient is one if one of the sets of variables differs from the other only by some combination of a rotation, reflection, scaling, or translation (i.e., if **x**_1_=**x**_2_**A**+**c**, where **A** is a square matrix for which **AA**^T^=*b***I** for some constant *b*>0 and the identity matrix **I**, and **c** is a constant vector of length *q*). As a consequence, the *RV* coefficient is invariant under rotation, translation and uniform scaling. Therefore, the *RV* coefficient does not depend on the choice of alignment of the landmark configurations relative to the coordinate system (but of course, it does matter how the two subsets of landmarks are aligned relative to each other by a Procrustes fit).

### The effect of Procrustes superimposition

Analyses of integration can use two different approaches to quantify the covariation between parts of a configuration of landmarks. One possibility is to analyze the shape of the configuration as a whole and to examine the covariation of parts within it. The alternative is to analyze the shapes of the parts separately as if they were entirely separate configurations and to assess the association between the different shapes. The difference between the two types of analysis is in how they treat the information about the connection between the subsets.

The first approach uses a single Procrustes fit for all landmarks jointly and then examines the covariance of subsets of landmarks within the overall configuration (I will call this the “simultaneous-fit” approach). It therefore explicitly considers the information about the connection of the subsets. It is possible that a portion of the covariation between subsets does not arise from simultaneous variation within the two subsets themselves, but stems from variation in the manner in which the subsets are connected. For this approach, the subsets of landmarks must be mutually exclusive, that is, each landmark can only belong to one subset.

The second approach, which treats the subsets as entirely separate configurations, uses two independent Procrustes fits to analyze the shapes of the subsets of landmarks (I will call this the “separate-subsets” approach). This approach ignores the anatomical connection of the two subsets, and therefore will record covariation between the subsets only if there are joint changes of shape within each subset. The connection between subsets can be taken into account by including landmarks that are on the boundary between adjoining anatomical units into both subsets. Because of the separate Procrustes fits, such overlap of the subsets of landmarks does not unduly inflate the estimates of covariation (but it does introduce a certain amount of redundant information). This approach ignores the information on the relative sizes of the different regions, which may be another feature of covariation, unless at least two landmarks are shared by the subsets.

**Fig. 4:**
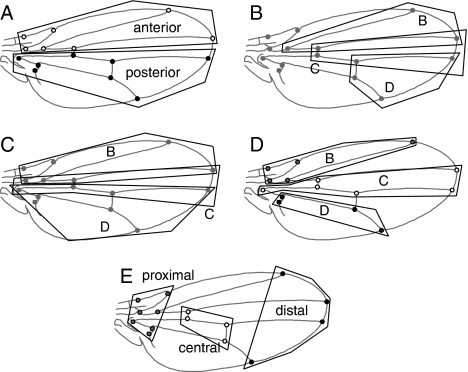
Possible subdivisions of the *Drosophila* wing. (A) The anterior and posterior compartments. (B) The division into wing sectors according to intervein areas (Birdsall et al. 2000; Zimmerman et al. 2000; Palsson and Gibson 2004;). (C) The extended version of the division into three sectors, covering all 15 landmarks. Note that the sectors B and D are the same as the anterior and posterior compartments. (D) Division into three mutually exclusive wing sectors. (E) Division into proximal, central, and distal regions.

To compare these two approaches directly, I subdivide the *Drosophila* wing into subsets of landmarks in several different ways (Fig. 4) and compute the *RV* coefficient for each pair of subsets. The example demonstrates that the results of the two types of analysis differ substantially (Table 1). For the subdivisions of the wing into mutually exclusive subsets, where the two approaches can be compared directly, the simultaneous Procrustes fit produces *RV* coefficients that are consistently higher, and often several times greater, than the ones obtained from the approach with separate Procrustes fits for the different subsets. Moreover, for the separate-subsets method, the *RV* coefficient appears to increase with the degree of overlap between the subsets of landmarks (compare Fig. 4B–D; Table 1, parts B–D). The *RV* coefficients tend to be somewhat higher for variation among individuals than for fluctuating asymmetry, although this trend is not entirely consistent. This difference between the two levels of variation is small in comparison to the difference between the two alternative methods.

**Table 1:** ***RV* coefficients and *P*-values from the corresponding permutation tests for different subdivisions of the *Drosophila* wing**

	Variation among individuals				Fluctuating asymmetry			
	Joint Procrustes fit		Separate Procrustes fits		Joint Procrustes fit		Separate Procrustes fits	
Comparison	*RV*	*P*	*RV*	*P*	*RV*	*P*	*RV*	*P*
(A) Anterior and posterior compartments								
Anterior and posterior	0.462	<0.0001	0.310	<0.0001	0.449	<0.0001	0.259	<0.0001
(B) Wing sectors according to Birdsall et al. (2000) and Zimmerman et al. (2000)								
Sectors B and C			0.172	<0.0001			0.240	<0.0001
Sectors B and D	0.357	<0.0001	0.149	<0.0001	0.291	<0.0001	0.023	0.45
Sectors C and D			0.272	<0.0001			0.402	<0.0001
(C) Extended wing sector scheme								
Sectors B and C			0.395	<0.0001			0.554	<0.0001
Sectors B and D	0.462	<0.0001	0.310	<0.0001	0.449	<0.0001	0.259	<0.0001
Sectors C and D			0.681	<0.0001			0.719	<0.0001
(D) Mutually exclusive wing sectors								
Sectors B and C	0.260	<0.0001	0.081	0.0038	0.168	0.0006	0.062	0.027
Sectors B and D	0.279	<0.0001	0.063	0.011	0.241	<0.0001	0.044	0.062
Sectors C and D	0.398	<0.0001	0.226	<0.0001	0.263	<0.0001	0.062	0.013
(E) Proximal, central, and distal regions								
Proximal and central	0.254	<0.0001	0.061	0.026	0.251	<0.0001	0.032	0.28
Proximal and distal	0.289	<0.0001	0.067	0.019	0.304	<0.0001	0.031	0.56
Central and distal	0.271	<0.0001	0.131	0.0001	0.201	<0.0001	0.009	0.82

The subdivisions of the wing referred to in parts A–E of the table are shown in the corresponding panels of Fig. 4. The tabled values are the RV coefficients between pairs of subsets (*RV*) and the corresponding *P*-values from permutation tests. Nonoverlapping subsets of landmarks were analyzed with separate as well as joint Procrustes fits. For overlapping sets of landmarks, only the analysis with separate Procrustes fits is feasible. The permutation tests with joint Procrustes fits included a Procrustes fit in each round of reshuffling the observations in the sets of variables.

### Statistical tests of covariation

The statistical significance of the covariation between sets of landmarks is usually established by means of a permutation test (e.g., Good 2000; Manly 2007;). To simulate the null hypothesis of complete independence between subsets, the observations in the different sets of landmarks are permuted randomly so that any association between sets is due to chance only. This procedure is repeated a large number of times, and each time a measure of covariation is computed and compared with the original value. The proportion of permutation rounds in which the measure of covariation matches or exceeds the original value is the significance level of the test.

The standard permutation test is feasible for testing the significance of covariation between sets of landmarks with the approach using separate Procrustes fits. The *RV* coefficient itself can be used as a test statistic. For each cycle of the permutation procedure, the observations in one of the subsets of landmarks are randomly reshuffled (i.e., the Procrustes coordinates of the configurations in the subset) and the *RV* coefficient of the resulting data set with the Procrustes coordinates of the other subset is then computed. The significance level of the test is the proportion of cases in which the *RV* coefficient computed for these modified data matches or exceeds the value obtained from the original data.

For the example of fly wings, this procedure was used to test the statistical significance of the *RV* coefficients computed from separate Procrustes fits (Table 1). For each test, 10,000 permutation rounds were used. As indicated by the low *P*-values, most of the associations between different wing regions are highly significant. The exception concerns the analyses of the proximal, central, and distal regions of the wing, where none of the three tests for fluctuating asymmetry indicates a significant association when analyzed with the approach of separate Procrustes fits (Table 1, part E).

When the simultaneous-fit method is used, however, this straightforward version of the permutation test of covariation is not feasible. Because the Procrustes superimposition finds an optimal fit for all the landmarks in the entire configuration jointly, it inevitably generates interdependence between different regions. This effect may be quite substantial, if the differences in *RV* coefficients between methods of simultaneous and separate Procrustes fits can be taken as an indication (Table 1). Therefore, an adjustment needs to be made to the permutation procedure so that it accounts for the effects of the Procrustes fit. This can be done by including a new Procrustes fit in every round of the permutation procedure (Klingenberg et al. 2003, 2004).

This modified procedure starts with the Procrustes coordinates, for which the *RV* coefficient between the landmark positions in the two regions is computed. Then the observations in one of the two sets are randomly exchanged, so that the association between sets is entirely by chance. After combining the two parts again, the newly assembled configurations will not be exactly in Procrustes superimposition because the parts do not quite “fit together”—in other words, the centroids (centers of gravity) will not match exactly, there will be slight variation in centroid size, and there also will be small differences in the overall orientation. To redress these, a new generalized Procrustes fit needs to be done. As a result, the coordinates of the combined configurations vary only in shape, but the Procrustes fit also results in a joint scaling, translation, and rotation that may induce a certain amount of covariation between the parts of the configuration. The *RV* coefficient can then be computed and compared with the value obtained in the original data. The landmark coordinates after reshuffling and re-fit are the appropriate basis of comparison for test of independence because the random permutation of the coordinates from one part of the configuration has eliminated systematic covariation and the Procrustes re-fit takes into account the covariation induced by the superimposition itself. This procedure is repeated for every round of the permutation test, each time using the landmark coordinates from the original Procrustes fit as the starting data.

A further modification of this test is required for analyses of covariation in fluctuating asymmetry (Klingenberg et al. 2003). Because the overall configuration is required for the scaling and rotation steps of the Procrustes fit, it would be erroneous to use just the left–right asymmetries in the Procrustes re-fitting procedure. For that reason, the overall mean shape is added to every vector of the asymmetries of landmark coordinates. Adding a constant in this manner has no effect on the covariation between the sets of landmarks, but it ensures that the re-fitting in each round of permutations is done correctly.

The permutation test with Procrustes re-fitting was applied to all comparisons of mutually exclusive sets of landmarks in the *Drosophila* wing. Most of the tests show significant covariation between the sets of landmarks (Table 1). For most analyses, the differences in *P* values between the tests using simultaneous or separate Procrustes fits are small, suggesting that there may not be a large difference between the two procedures. In a few cases, however, there are considerable differences between the results for the two test procedures for fluctuating asymmetry (Table 1, parts D and E). It appears that the difference between the two test procedures depends considerably on specific properties of the data. For instance, it is conceivable that in the subdivision into proximal, central, and distal subsets, which are fairly compact and distant from each other (Fig. 4E), the relative sizes and arrangement of subsets make a greater contribution to overall integration than in the other subdivisions.

### A measure of association for multiple sets of landmarks

If there are more than two sets of landmarks, the *RV* coefficient can be used to assess the strength of association between each pair of sets, but it does not provide an overall measure of association among all the subsets simultaneously.

I define a new measure of association among multiple sets of variables, the multi-set *RV* coefficient, as the average of all pair-wise *RV* coefficients between sets: 
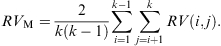


In this formula, *k* is the number of sets of variables and the notation *RV*(*i, j*) is used to designate the *RV* coefficient for the sets *i* and *j*.

The multi-set *RV* coefficient can be tested against the null hypothesis of independence among all sets of variables by a permutation approach that extends the one outlined above for two sets of variables. This can be considered as an overall test of integration among the subsets. The test starts with the Procrustes coordinates of the landmarks, from which the observed value of *RV*_M_ is computed (for analyses of fluctuating asymmetry with a simultaneous Procrustes fit for all subsets, the mean shape for all configurations is added to each of the individual right–left differences). The null hypothesis of independence among the sets of variables is simulated by randomly permuting observations in each subset (one subset can be left in the original order). If a separate Procrustes fit is used for each subset of landmarks, then the *RV*_M_ under the null hypothesis can be computed directly and compared with the original value. Conversely, if a simultaneous Procrustes fit for all subsets is used, then a new overall Procrustes fit of the permuted data is necessary in each permutation round before computing the *RV*_M_ value. This procedure is repeated a large number of times, and the significance level of the test is the proportion of rounds in which the *RV*_M_ value for the permuted data matches or exceeds the value observed originally.

The multi-set *RV* coefficients for the subdivisions of the fly wing into three nonoverlapping subsets (Table 2) are, by definition, equal to the averages of the corresponding *RV* coefficients for pairs of landmark sets (Table 1), and therefore the results are necessarily similar. All but one of the permutation tests indicated that the associations among sets of landmarks were statistically significant (Table 2), which also is in overall agreement with those for the pair-wise analyses of landmark sets (Table 1).

**Table 2:** **Multi-set *RV* coefficients and *P* values from the corresponding permutation tests for different subdivisions of the *Drosophila* wing**

	Variation among individuals				Fluctuating asymmetry			
	Joint Procrustes fit		Separate Procrustes fits		Joint Procrustes fit		Separate Procrustes fits	
Comparison	*RV*_M_	*P*	*RV*_M_	*P*	*RV*_M_	*P*	*RV*_M_	*P*
(D) Mutually exclusive wing sectors								
All three subsets	0.312	<0.0001	0.124	<0.0001	0.224	<0.0001	0.056	0.0013
(E) Proximal, central and distal regions								
All three subsets	0.271	<0.0001	0.087	<0.0001	0.252	<0.0001	0.024	0.63

Only the subdivisions of the wing into three nonoverlapping subsets are considered (cf. Fig. 4, D, E, Table 1). The tabled values are the squared multi-set trace correlations among subsets (*RV*_M_) and the corresponding *P*-values from permutation tests. The permutation procedure for joint Procrustes fits included a new Procrustes fit in each round of permutation.

## LOCATING BOUNDARIES BETWEEN MODULES

The *RV* coefficient or multi-set *RV* coefficient can be used to quantify covariation in the context of testing a hypothesis about the boundary between modules (Fig. 1). If the hypothesized partition coincides with the true subdivision of the configuration into modules, the *RV* coefficient between subsets should be lower than is expected for alternative partitions of the configuration into subsets of landmarks.

In the following analyses, the comparisons are limited to alternative partitions that consist of subsets containing the same numbers of landmarks as the hypothesized modules. The primary aim of this limitation is to ensure that the comparisons are “fair.” Holding constant the numbers of landmarks in the subsets maintains a relatively homogeneous behavior across the partitions that are being compared, and avoids potential artifacts due to extreme sizes of the subsets. For instance, it is often possible to obtain very weak covariation between sets by choosing a partition that separates a single landmark from all the others. Moreover, the limitation to partitions with the same sizes of subsets also limits the number of comparisons that need to be made, and thus makes the comparison computationally feasible even with relatively large numbers of landmarks.

### Comparing alternative partitions

The most straightforward approach to evaluate a hypothesis of subdivision of a configuration of landmarks into two modules is to compute the *RV* coefficient for all possible partitions into subsets of the appropriate sizes. If the hypothesis of modularity holds, the *RV* coefficient for the partition according to the hypothesis should be the lowest value, or it should at least be near the lower extreme of the distribution of *RV* coefficients for all of the partitions. The *RV* coefficients are computed from Procrustes coordinates resulting from the simultaneous Procrustes fit for all landmarks together.

For partitioning a configuration of *m* landmarks into two subsets of *k* and *m*−*k* landmarks, it is feasible to enumerate all possible partitions as long as *m* is not too large. The number of such partitions is the number of combinations of *k* out of *m* objects, that is, 
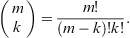


In the special case where both sets contain the same number of landmarks (i.e., *m*=2*k*), this number needs to be divided by two because each possible partition is listed twice in different orders, for example (1, 2) and (3, 4) as well as (3, 4) and (1, 2). Overall, the number of possible partitions increases very rapidly with the total number of landmarks in the configuration. For instance, for the division of 10 landmarks into two subsets of five, there are 126 possible partitions, whereas there are 92,378 partitions of 20 landmarks into subsets of 10 each.

The complete enumeration of all possible partitions therefore may not be computationally feasible for landmark configurations with more than about 20 landmarks. In this case, random partitions of the configuration into subsets of the appropriate number of landmarks can be used instead. I recommend a number of random partitions in the order of 10,000 for the comparison, which should provide a reasonable characterization of the distribution of the *RV* coefficient. A large number is needed because the primary interest concerns the left tail of the distribution.

**Fig. 5:**
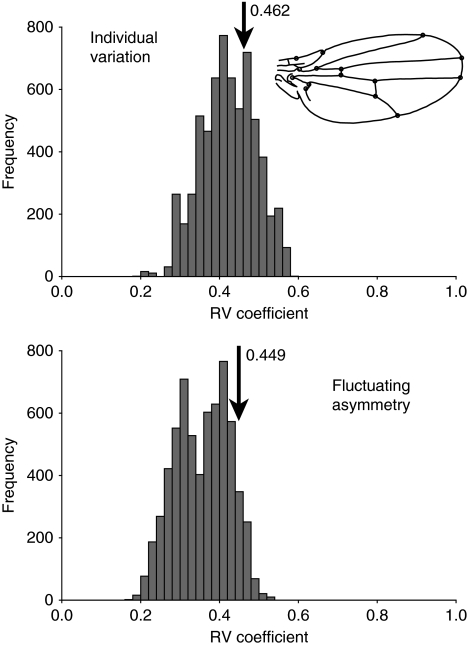
Histograms of the squared trace correlations between all possible partitions of the *Drosophila* wing. The values of the squared trace correlation between the subsets of landmarks in the anterior and posterior compartments are indicated by arrows.

I have applied this approach to the *Drosophila* wing example to re-evaluate the hypothesis that the anterior and posterior compartments are modules (Figs. 2 and 4A). The configuration of 15 landmarks is therefore subdivided into subsets of seven and eight landmarks. Because the total number of different partitions into subsets of seven and eight landmarks is 6435, it was feasible to enumerate them completely and to compute the *RV* coefficients for all of them (Fig. 5). For both variation among individuals and fluctuating asymmetry, the *RV* coefficient obtained for the partition into anterior and posterior compartments (arrows in Fig. 5) is to the right of the mode of the distribution of the values for all possible partitions. For individual variation, 4374 of the partitions result in a lower *RV* coefficient, and for fluctuating asymmetry, 5916 partitions yield a lower value, indicating clearly that the observed value is not in the left tail of the distribution. This means that the covariation between the anterior and posterior compartments is not weaker than it would be expected for a random partition of the landmark configuration. This result is evidence against the hypothesis that the anterior and posterior compartments are separate modules.

**Fig. 6:**
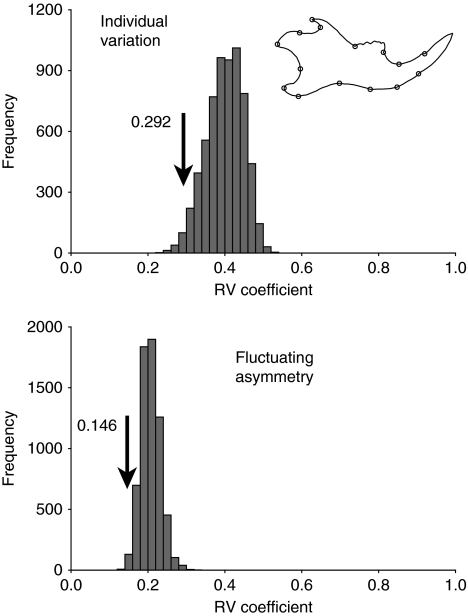
Histograms of the squared trace correlations for all possible partitions of the mouse mandible. The trace correlations between the alveolar region and the ascending ramus (indicated by arrows) are the lowest values observed for any of the 6435 partitions of the configuration of 15 landmarks into subsets of seven and eight landmarks.

A quite different result emerges for the data set of mouse mandibles, where the hypothesis is a subdivision into two modules, the alveolar region and ascending ramus (Fig. 3). This is also a subdivision of the whole configuration into subsets of seven and eight landmarks, and therefore is directly comparable to the fly wing example. The *RV* coefficients between subsets were computed for all 6435 alternative partitions for both the variation among individuals and fluctuating asymmetry (Fig. 6). For individual variation, only 113 partitions result in an *RV* coefficient that is lower than the one observed for the subdivision into alveolar region and ascending ramus, and for fluctuating asymmetry, there are only 22 such partitions. Accordingly, the *RV* coefficient for the subdivision is clearly near the lower extreme of the distribution of *RV* coefficients (arrows in Fig. 6). This result is consistent with the hypothesis that the alveolar region and ascending ramus of the mouse mandible are distinct modules.

## SPATIAL CONTIGUITY OF MODULES

A possible objection against the procedure outlined above is that the set of all possible partitions of a landmark configuration is not a biologically realistic base of comparison. This assemblage includes many subdivisions where one or both subsets of landmarks are not contiguous, but are composed of landmarks in different parts of configuration that are spatially separated. Depending on the biological context of a study, such spatially disjoint sets of landmarks may not be plausible candidates for modules.

If the internal integration of modules relies on tissue-bound interactions among their parts, modules cannot be divided into components that are spatially separated from each other, because such a separation would prevent interactions between them. For instance, developmental fields, which are often the precursors of morphological modules, need to be spatially contiguous because they are defined by signaling interactions among nearby cells (e.g., Davidson 1993, 2001; Carroll et al. 2001; Kornberg and Guha 2007). In biological contexts such as this, it may therefore be preferable to consider a set of landmarks as a possible candidate for a morphological module only if it is spatially contiguous.

To study modularity in this context, it is necessary to establish a procedure that limits comparisons to just those partitions for which the subsets of landmarks are spatially contiguous. This, in turn, requires a definition of spatial contiguity that is computationally tractable. I use an approach that is based on the theory of graphs, which can easily be incorporated into the combinatorial framework of this analysis of modularity. Several graph-based approaches for studies of integration were briefly discussed by Chernoff and Magwene (1999), whereas Gabriel and Sokal (1969) used graphs as a criterion of spatial contiguity in geographic analysis.

### A definition of contiguity using adjacency graphs

**Fig. 7:**
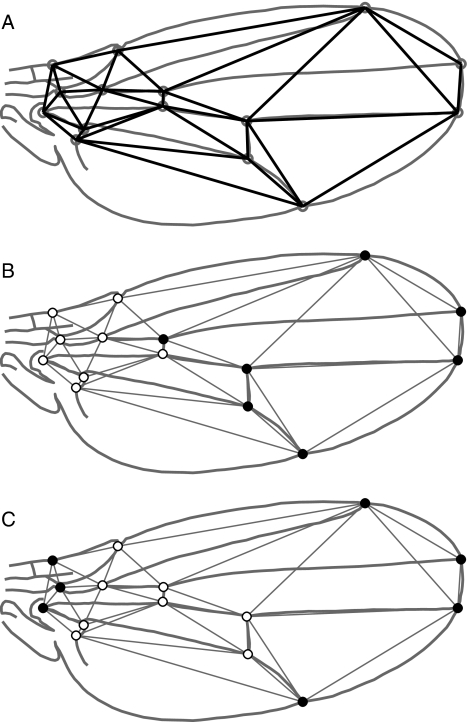
Definition of spatial contiguity for sets of landmarks. (A) An adjacency graph for the *Drosophila* wing. The edges of this graph connect neighboring landmarks. This adjacency graph has been obtained as the Delaunay triangulation (e.g., de Berg et al. 2000) of the landmark positions in the mean shape. A set of landmarks is said to be contiguous if every one of its landmarks is connected directly or indirectly to all other landmarks of the set by the edges of this graph. (B) An example of a contiguous set of landmarks (black circles). Within this set, all landmarks are connected to each other either directly or indirectly via other landmarks of the set. (C) An example of a set of landmarks that is not contiguous. It consists of one group of three landmarks at the base of the wing and another group near the wing tip (solid black circles), which are separated from each other by landmarks belonging to the other set (open circles).

A definition of spatial contiguity in sets of landmarks requires a way to assess whether any two landmarks in the configuration are neighbors. I offer a definition of contiguity that is based on adjacency graphs, in which the nodes represent the landmarks in the configuration and the edges connect neighboring landmarks (Fig. 7A).

I start by providing a definition of contiguity for a single set of landmarks. *A set of landmarks is spatially contiguous if every landmark of the set is connected by the edges of the adjacency graph to every other landmark in the set either directly or indirectly through other landmarks that also belong to the set*. This definition ensures that it is possible to move between any two landmarks of the set along the edges that connect landmarks belonging to the set. For instance, in Fig. 7B, the set of landmarks marked by solid black dots and the set of landmarks marked by hollow dots are both contiguous. In contrast, the set of solid black dots in Fig. 7C is not contiguous because it is divided into two parts at the base and the tip of the fly wing, and any movement between the two parts along the edges of the adjacency graph must pass through at least one landmark of the other set (landmarks marked by hollow dots).

The definition of a spatially contiguous set of landmarks can be extended to a definition of a partition of the entire configuration. *A partition is spatially contiguous if it divides a configuration into sets of landmarks that are all spatially contiguous themselves*. This means that a partition is only considered spatially contiguous if all the resulting sets of landmarks are spatially contiguous. For example, the partition of landmarks into two sets in the proximal and distal parts of the wing form a spatially contiguous partition (Fig. 7B). In contrast, a division into a central region and a second set of landmarks at the base and the tip of the wing is not spatially contiguous (Fig. 7C; but note that this would be a spatially contiguous partition into *three* sets of landmarks).

### Obtaining adjacency graphs

As a strictly geometric criterion to define adjacency of landmarks, I use the Delaunay triangulation of the landmark positions in the average configuration (e.g., de Berg et al. 2000, chapter 9). The Delaunay triangulation divides a configuration of points into nonoverlapping triangles, so that none of the points lies inside the circumcircle of one of the triangles. As a result, the triangulation avoids very long and narrow triangles as far as it is possible, given the whole configuration. The connections through the edges of the triangulation can therefore serve as a criterion to determine which landmarks are next to each other in the configuration (e.g., Fig. 7A).

It is possible to extend this scheme to three dimensions because there is an equivalent to the Delaunay triangulation in the plane. Such a Delaunay tessellation in three dimensions divides the volume inside the convex hull of the landmark configuration into nonoverlapping tetrahedra so that the sphere that passes through the four vertices of each tetrahedron does not contain any of the other landmarks. The edges of this tessellation can be used to define the adjacency of landmarks, just as in the two-dimensional triangulation.

**Fig. 8:**
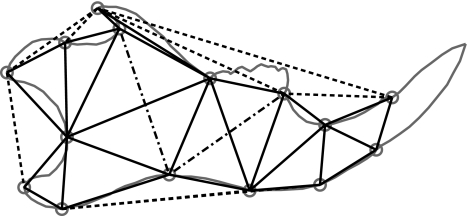
Possible problems with the Delaunay triangulations for configurations with a complex outline. The outline of the mouse mandible is indented in regions such as the space between the incisor and molar teeth or between the muscle attachment processes. Some of the edges of the Delaunay triangulation are therefore outside the contour of the mandible (dashed lines). These may be omitted for the consideration of spatial contiguity. For each quadrilateral of neighboring landmarks, the Delaunay triangulation contains just one of the two diagonals; the second diagonal may be added to the adjacency graph (dot-dashed lines; note that this has not been done for all possible quadrilaterals).

Because the Delaunay triangulation uses only the geometry of the mean shape, it cannot take into account any anatomical or other biological factors. To obtain an adjacency graph that is biologically meaningful, the investigator may decide to modify the graph by eliminating or adding some edges. For instance, it may be necessary to modify the edges of the adjacency graph if the outline of the configuration has a complex shape. The Delaunay triangulation applies to the entire region within the convex hull surrounding the landmarks. This is appropriate if the structure represented by the landmark configuration is convex itself, as is the case for the fly wing (Fig. 7). If the structure has concave regions, however, such as the indentations between the attachment processes of the mouse mandible (Fig. 3), the Delaunay triangulation may have edges that are located outside of the structure and may not correspond to biological links between landmarks. These edges can be removed manually to limit the criterion for spatial contiguity to the regions inside the contour of the structure (dashed lines in Fig. 8). There may be other reasons for modifying the adjacency graphs. The Delaunay triangulation may not always contain all the links that are relevant from a biological perspective. For instance, for quadrilaterals of nearby landmarks, the triangulation will contain only one of the two diagonals, even if both are nearly of the same length. It may therefore be desirable to add some of those diagonals to the adjacency graph (dot-dashed lines in Fig. 8).

A different problem applies particularly to studies of structures such as skulls, where the landmarks often are collected on the surface only. Moreover, major anatomical features such as the cranial vault and parts of the face are also organized by developmental interactions that take place near the surface of the developing head. It is therefore questionable whether the relationships among landmarks should be defined by geometric proximity in the skull volume, which may link landmarks of the cranial base with those of the skull vault. Extensive alterations of the adjacency graph may be necessary in situations like this.

Overall, the Delaunay triangulation should be taken only as an initial approximation of the adjacency graph, which may require substantial changes. The Delaunay triangulation is useful as a starting point, however, because it can be easily implemented in computer programs with widely available algorithms (e.g., de Berg et al. 2000, chapter 9). For instance, the MorphoJ software (Klingenberg 2008–2009) implements the Delaunay triangulation in two or three dimensions, but also provides the user with an interface for modifying the adjacency graphs.

### Application

**Fig. 9:**
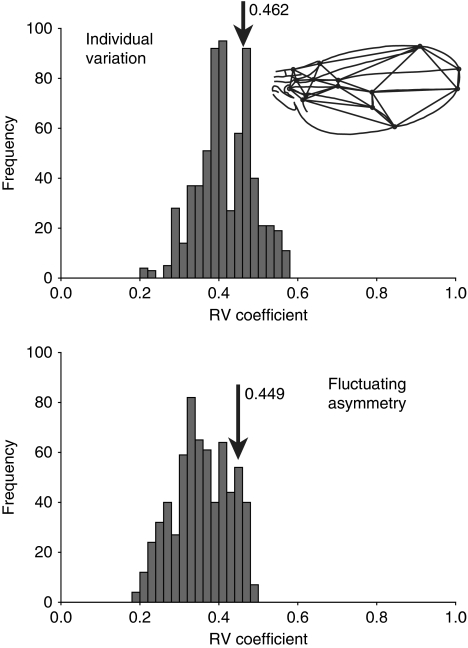
Histograms of the squared trace correlations for those partitions of the *Drosophila* wing that produced spatially contiguous subsets of landmarks. The values of the squared trace correlation between the subsets of landmarks in the anterior and posterior compartments are indicated by arrows.

Of the 6435 partitions of the landmarks of the fly wing into sets of seven and eight landmarks, 655 divided them into two sets that were both spatially contiguous. The distribution of the values of the *RV* coefficient between the subsets of landmarks covers a similar range as the distribution of all possible partitions (Fig. 9). For the variation among individuals, 457 of these spatially contiguous partitions had *RV* coefficients that were lower than the one between the anterior and posterior compartments. Likewise, for fluctuating asymmetry, 578 spatially contiguous partitions had a *RV* coefficient lower than that between the two compartments. The observed correlations are thus in the top one-third of the distribution and clearly not near the lower end of the distribution, as it would be expected if the two compartments were distinct morphological modules. A comparison of the histograms in Figs. 5 and 9 shows that the limitation to only the spatially contiguous partitions did not lead to any major changes in the distribution of *RV* coefficients for either individual variation or fluctuating asymmetry.

**Fig. 10:**
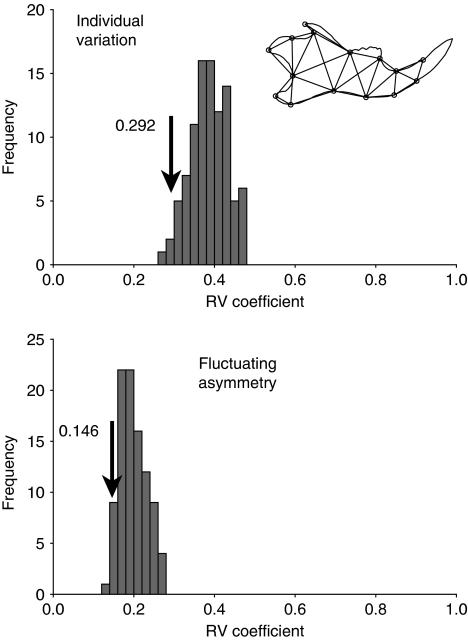
Histograms of the *RV* coefficients for those partitions of the mouse mandible that produced spatially contiguous subsets of landmarks. The values of the *RV* coefficients between the subsets of landmarks in the alveolar region and ascending ramus are indicated by arrows.

For the mouse example, only 95 of the 6435 partitions of the configuration into two subsets of seven and eight landmarks resulted in two contiguous subsets. For the variation among individuals, only one of those 95 partitions resulted in an *RV* coefficient lower than that observed for the hypothesized partition into the alveolar part and ascending ramus (Fig. 10). For fluctuating asymmetry, only two of the 95 contiguous partitions produced a lower *RV* coefficient. This result underscores the previous finding that the RV coefficient for the partition into the hypothesized modules is near the lower end of the spectrum for the entire range of partitions into seven and eight landmarks (cf. Fig. 6).

## MORE THAN TWO MODULES

The method of comparing alternative partitions of a landmark configuration can also be applied when a hypothesis specifies more than two subsets of landmarks. In this case, the multi-set *RV* coefficient is used to quantify the covariation among subsets.

A difficulty with this method is that the number of possible partitions increases very rapidly with the total number of landmarks. For a configuration of *m* landmarks that is subdivided into *k* subsets of *m_i_* landmarks (*i*=1, …, *k*, with all *m_i_* summing up to *m*), the total number of possible partitions is 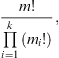
if all the *m_i_* are different from each other. If some of the sets have the same number of variables, then this number should be divided by the factorial of the number of equal-sized sets to avoid counting the same partition multiple times with the subsets in different orders (e.g., for a subdivision into three equal subsets, the division is by 3==6).

For a configuration of 15 landmarks, there are 630,630 possible partitions that produce three subsets of four, five, and six landmarks (e.g., Fig. 4D). For a subdivision of the same configuration into three unequal subsets of seven, four, and four landmarks (e.g., Fig. 4E), there are still 225,225 possible partitions. Computing the multi-set *RV* coefficient for all these partitions involves a substantial computational effort, and it may therefore be reasonable to use a sufficiently large number of random partitions of the landmarks. For most purposes, 10,000 random partitions should be sufficient.

For the case study of the *Drosophila* wings, I ran analyses for two different subdivisions of the landmarks into three subsets (see Fig. 4, D and E and Table 2). The first example is a subdivision along the anterior–posterior axis of the wing into three subsets of four, five, and six landmarks (Fig. 4D). Of the total 630,630 partitions of the wing into three subsets of four, five, and six landmarks, the *RV*_M_ value is less than that for the original partition (Table 2) in 195,180 and 95,322 cases for individual variation and fluctuating asymmetry, respectively. Neither of these *RV*_M_ values is therefore near the left extreme of the distribution, which is evidence against the hypothesis that the three wing sectors in Fig. 4D are morphological modules.

The second example concerned the division of the *Drosophila* wing into subsets of seven, four, and four landmarks corresponding to proximal, central, and distal sectors (Fig. 4E). There are 225,225 possible partitions of 15 landmarks into subsets of seven, four, and four landmarks. Of these, 23,555 have *RV*_M_ values for the variation among individuals that are less than the hypothesized modules, and 73,235 partitions have lower *RV*_M_ values for fluctuating asymmetry. Again, the *RV*_M_ values for the partition according to the a priori hypothesis are not unusually low by comparison with the distribution of *RV*_M_ values for alternative partitions. Therefore, a principal expectation of the hypothesis of modularity is not met by these data.

### Spatial contiguity of multiple modules

Just as for a partition into two subsets, it is also possible to limit the comparison to spatially contiguous partitions with more than two subsets. This can result in a drastic reduction of the number of partitions because only a relatively small fraction of the possible partitions are spatially contiguous. For configurations with a moderate number of landmarks and relatively few subsets, it is therefore usually feasible to use a complete enumeration of contiguous partitions.

For the subdivision of the *Drosophila* wing into anterior, middle, and posterior sectors (Fig. 4D), the set of landmarks in the middle sector is not spatially contiguous with reference to the adjacency graph in Fig. 7A. Therefore, this partition as a whole is not spatially contiguous, and thus it is not meaningful to make the comparison with alternative partitions that are limited to be spatially contiguous. A possibility would be to alter the adjacency graph so that all three sectors are spatially contiguous.

For the partition of the wing into proximal, central, and distal regions (Fig. 4E), limiting the comparison to the spatially contiguous partition reduces the number of comparisons from 225,225 to 1414. For these spatially contiguous 1414 partitions, the *RV*_M_ values were less than the one for the partition into proximal, central, and distal regions in 241 partitions for individual variation and in 523 partitions for fluctuating asymmetry. Therefore, this result is inconsistent with the hypothesis that the three regions are separate modules, because a large proportion of randomly chosen partitions of the landmark configuration into contiguous subsets yield weaker covariation among subsets. Overall, for both individual variation and fluctuating asymmetry, this analysis is consistent with the alternative that the *Drosophila* wing is a single, fully integrated unit.

## THE INFLUENCE OF ALLOMETRY

Allometry is the influence of size on shape (and other organismal properties), and can have a major effect on patterns of integration, and therefore on the detection of modularity. Because the effects of size affect all parts of the entire organism jointly, they can produce global integration throughout the whole landmark configuration under study and may obscure a possible modular structure. The balance between modularity and such integrating processes may account in part for the finding that modularity in empirical data sets is not an “all-or-nothing” phenomenon, but that there is a gradation of degrees of integration and modularity (Klingenberg et al. 2003, 2004).

The effects of allometry can be addressed in a straightforward manner by first performing a multivariate regression of shape on size to characterize allometry (e.g., Monteiro 1999). The residuals from this regression represent the shape variation after subtracting the effects of allometry. Therefore, the analyses outlined in this article can be carried out using the covariance matrices of these residuals to analyze modularity after removing the influence of allometry.

For the example of the *Drosophila* wing, a multivariate regression of individual variation of shape on centroid size shows that allometry is statistically significant (permutation test with 10,000 random permutations, *P*<0.0001). Centroid size accounts for 16.9% of the Procrustes variance, and thus allometry is responsible for an appreciable part of the shape variation in the sample. Accordingly, the correction for allometry was made by computing the *RV* coefficients from the covariance matrix of the residual from a multivariate regression of the Procrustes coordinates on centroid size. Even after this correction, the *RV* coefficient for the covariation between the landmarks in the anterior and posterior compartments is 0.462, just as it is for the uncorrected covariance matrix (Table 1). In the comparison with alternative partitions of the landmarks into subsets, this *RV* coefficient is to the right of the mode of the distribution of corrected *RV* coefficients for the alternative partitions of the landmarks.

The same allometry correction can be applied to the asymmetry component as well, by taking the residuals from a multivariate regression of the signed asymmetries of shape on the signed asymmetry of centroid size. This correction removes the component of fluctuating asymmetry of shape that is related to fluctuating asymmetry of size (and presumably is a developmental consequence of it). This regression is statistically significant (permutation *P*<0.0001) and accounts for 7.3% of the fluctuating asymmetry of shape. The *RV* coefficient between landmarks in the anterior and posterior compartments is 0.425 for this allometry-corrected asymmetry, which is slightly less than the original value of 0.449. Because the allometry correction results in a reduction of the *RV* coefficients for most partitions of the landmarks in the fly wing, however, the *RV* coefficient for the anterior and posterior compartments is still higher than the values for the majority of possible partitions. In sum, the correction for allometric effects produces only small changes the covariation of landmarks between the anterior and posterior compartments of the *Drosophila* wing.

The effect of correcting for allometry is more apparent for the data set of mouse mandibles. The allometric regression accounts for 18.4% of the individual variation of shape (permutation *P*<0.0001). The correction for allometry reduces the *RV* coefficient between the alveolar region and ascending ramus from 0.292 to 0.219, and only seven of the 6435 possible partitions have a lower *RV* coefficient (none of the 95 contiguous partitions have a lower *RV* coefficient). For fluctuating asymmetry, allometry accounts for 6.1% of the variation (permutation *P*<0.0001). The allometry correction reduces the *RV* coefficient between the two regions from 0.146 to 0.134, and only nine partitions have a lower RV coefficient (only one of the contiguous partitions has a lower *RV* coefficient). Overall, the allometry correction reduces covariation for the mouse mandibles and accentuates the relative independence of the alveolar region and ascending ramus.

## DISCUSSION

This article has introduced new methodology for investigating morphological integration and modularity in configurations of landmarks. In geometric morphometrics, morphological modules manifest themselves as groups of landmarks that are minimally correlated with other such groups. It is thus possible to evaluate hypotheses of modularity directly by comparing the strength of covariation for alternative partitions of landmarks into subsets (Fig. 1). The *RV* coefficient (Escoufier 1973; Robert and Escoufier 1976;) and its multi-set generalization can be used as measures of covariation among subsets of landmarks. Depending on the biological context of a study, the investigator may also require that morphological modules are spatially contiguous. Adjacency matrices offer an operational criterion to define spatial contiguity of subsets of landmarks. These methods are complementary to other methods for finding modules and fitting models of covariance structure (Monteiro et al. 2005; Goswami 2006a,b; Mitteroecker and Bookstein 2007; Márquez 2008; Zelditch et al. 2008;). I have illustrated these methods with two case studies concerning the variation among individuals and fluctuating asymmetry in the *Drosophila* wing and the mouse mandible.

### Quantifying covariation in landmark configurations

The analysis of covariation in fly wings revealed substantial differences in *RV* coefficients, depending on whether the Procrustes fit was done jointly for the whole landmark configuration or separately for each part (Tables 1 and 2). Unsurprisingly, the *RV* coefficients were much higher for the joint Procrustes fit than for separate Procrustes fits. For the majority of the analyses, the outcomes of the permutation tests were similar, but for some of the *RV* coefficients, the test for the procedure with separate Procrustes fits was not significant.

The difference between the two approaches may partially explain the contrasting results of different published studies. Whereas analyses based on joint Procrustes fits have underscored the integration across the *Drosophila* wing (Klingenberg and Zaklan 2000), studies based on separate Procrustes fits have reported low phenotypic and genetic correlations and differences in the genetic architecture of principal components of shape for different subsets of landmarks (Birdsall et al. 2000; Zimmerman et al. 2000; Palsson and Gibson 2004; Dworkin and Gibson 2006;). The results obtained here (Tables 1 and 2) show that such discrepancies can result from differences in the analyses used, rather than contradictions of the data.

Both approaches have been used extensively for analyses of integration in other study systems. For rodent mandibles, some authors used the simultaneous-fit method (Klingenberg et al. 2003; Márquez 2008; Zelditch et al. 2008;) and others used separate Procrustes fits (Monteiro et al. 2005). Likewise, some studies of integration in the skulls of humans and other mammals were based on the simultaneous-fit approach (Bookstein et al. 2003; Goswami 2006a,b; Mitteroecker and Bookstein 2008;), whereas others used separate subsets (Bastir and Rosas 2005, 2006; Cardini and Elton 2008).

These differences raise the question of which method should be used for future studies. The differences in results reflect the differences in the information considered by the two approaches. Whereas the method of separate Procrustes fits exclusively considers the shapes of parts, each taken in isolation, the analysis based on a joint Procrustes fit also concerns covariation in the relative sizes and arrangement of the different parts. Therefore, the choice of the method should be based on whether the primary interest is in the covariation of individual parts or in the joint variation of the overall structure as a whole. The estimated covariation in the simultaneous-fit method can be criticized for being “inflated” by the joint Procrustes fit, whereas it can be seen as a disadvantage that the separate-subsets method “misses” some features of the landmark configuration, namely the relative sizes and positions of the subsets. There is no absolute “right” or “wrong” in the choice of these methods, and investigators should choose the method that is most suitable for the context of a particular study, or present the results for both approaches. Evidently, comparisons between different studies need to take into account the difference between the two approaches.

### Evaluating hypotheses of modularity

The analyses of the two example data sets yielded contrasting results. The analysis of the *Drosophila* wing indicates that anterior and posterior compartments (Fig. 2) are not separate modules. The covariation between the landmarks in the two compartments is not any weaker than it would be expected for an arbitrary partition of the wing into two subsets of landmarks, regardless of whether all alternative partitions are considered or just the spatially contiguous ones (Figs. 5 and 9). Strong covariation within modules and relative independence between modules are a defining criterion for modularity, and this expectation is clearly not met for the fly wings.

This confirms the results of an earlier study, which used principal component analyses and partial least squares analyses to compare the patterns of covariation of landmarks throughout the entire wing with the patterns of covariation between the two compartments (Klingenberg and Zaklan 2000). Because the two analyses revealed congruent patterns, they were consistent with an extreme model of integration in which the entire wing is a single, fully integrated module. The present analyses extend these findings by considering additional subdivisions of landmarks along the proximal–distal or anterior–posterior axes of the wing (Fig. 4, Tables 1 and 2). All of these subdivisions showed fairly strong and statistically significant covariation between parts for both individual variation and fluctuating asymmetry, and none of the subdivisions that were considered resulted in a degree of covariation that was lower than what might be expected for a random partition of the landmarks. Therefore, these results are consistent with the model of the fly wing as a single, fully integrated module. The weak effect of the correction for allometry suggests that allometry is not the primary factor responsible for integration in the fly wing.

In contrast to the strong integration in the *Drosophila* wing, there is evidence for modularity in the mouse mandible. The results of the analyses presented in this article conform to the hypothesis that the alveolar region and ascending ramus are separate modules (Fig. 3; Atchley et al. 1985; Leamy 1993; Cheverud et al. 1997; Mezey et al. 2000; Ehrich et al. 2003; Klingenberg et al. 2003, 2004). Both for variation among individuals and for fluctuating asymmetry, the *RV* coefficients for this subdivision are consistently among the lowest of any possible partition of the configuration (Figs. 6 and 10). This result strengthens the findings from an earlier analysis of the same data set with only a limited number of alternative partitions (Klingenberg et al. 2003). A similar approach, when applied to the variation of the effects of quantitative trait loci, produced a somewhat ambiguous result, which may have resulted from the limited sample size of 33 loci (Klingenberg et al. 2004).

The *RV* coefficients for all possible partitions of the mouse mandible vary within a fairly narrow range, particularly for fluctuating asymmetry (Figs. 6 and 10). Even though the partition into the two hypothesized modules has one of the very lowest *RV* coefficients, the covariation for other partitions is not drastically higher. This confirms the previous results that modularity in the mandible can be a matter of degrees (Klingenberg et al. 2003, 2004). Moreover, other studies have considered subdivisions of rodent mandibles into more than two parts and found support for such more complex models of modularity as well (Monteiro et al. 2005; Márquez 2008; Zelditch et al. 2008;).

This example illustrates that the comparison of the *RV* coefficient for the partition of interest to the distribution of *RV* coefficients for the alternative partitions can provide more information than the *P*-value from a statistical test would. The proportion of partitions for which the *RV* coefficient is less than or equal to the *RV* value for the partition of interest, which can be interpreted as the analog of such a *P*-value, is one piece of information that can be obtained. Other information, such as the range of *RV* coefficients in the possible partitions, is also critically important for interpreting the patterns of modular variation in a landmark configuration.

### Spatial contiguity of modules

This article presents an operational approach for limiting the comparisons of alternative partitions to those that are spatially contiguous. Spatial contiguity is relevant in the context of many morphological studies (e.g., Chernoff and Magwene 1999). First, if morphological modules are to be coherent anatomical units, spatial contiguity is an important property defining their coherence and individuality as units (this is different, e.g., for functional modules, which conceivably can consist of several anatomically disjoint parts that interact in performing a function; Breuker et al. 2006). Moreover, if the internal integration of morphological modules originates from tissue-bound developmental interactions within their precursors, they are likely to relate to spatially defined developmental units such as embryonic fields (e.g., Davidson 1993; Gilbert et al. 1996; Carroll et al. 2001; Kornberg and Guha 2007;). For analyses of modularity in these contexts, is therefore reasonable to limit the comparisons to partitions that divide a landmark configuration into subsets that are all spatially contiguous.

This restriction to spatially contiguous partitions also substantially reduces the number of partitions for which the covariation among sets of landmarks needs to be quantified, and therefore diminishes the computational effort required. For the subdivision of the *Drosophila* wing into two subsets, just over 10% of the possible partitions were spatially contiguous. For the divisions into three subsets, this proportion was <1%. The specific proportions depend on the number and sizes of subsets and on the arrangement of landmarks, and will therefore differ from one data set to another. Nevertheless, it is clear that limiting the comparisons to spatially contiguous partitions can slow the explosive growth of the number of possible partitions with increasing numbers of landmarks in the configuration and increasing numbers of subsets. Analyses of modularity with this approach are therefore computationally feasible even with substantially more landmarks than were used in this article.

The adjacency graph is crucial for this definition of spatial contiguity in configurations of landmarks. The Delaunay triangulation is usually a reasonable starting point and may be directly usable as the adjacency graph, such as the example of fly wings (Fig. 7). It is important to note, however, that the Delaunay triangulation only takes into account geometric information and cannot take into account the anatomical and other biological details that are relevant for the connectivity among landmarks in the context of a particular study (see also Chernoff and Magwene 1999). In the majority of applications, therefore, it is to be expected that the investigator needs to modify the graph. These changes can involve removing edges of the triangulation that are outside the outline of the structure or inserting additional edges between landmarks, such as the second diagonal in quadrilaterals of landmarks (Fig. 8). These alterations demand some biological judgment by the investigator and will depend on the context of the study.

### Allometry and other external factors

Allometry, the effect of size on shape, is expected to have a simultaneous effect on all parts of a structure or even on the whole organism. Accordingly, it is expected to exert an integrating influence on morphological structures and thereby to counteract modularity. Similarly, multiple parts of an organism may respond jointly to environmental changes, and phenotypic plasticity may thus also act as an integrating factor. If modularity is of primary interest, it is thus reasonable to correct for the effects of allometry or other such factors.

I used regression to correct for effects of size, that is, the analysis of modularity uses the covariance matrix of the residuals from a regression of the shape variables on centroid size (e.g., Loy et al. 1998; Monteiro 1999;). For the two data sets used in this article, this allometric correction had different effects. For the *Drosophila* wings, there was little effect of the correction, whereas a somewhat clearer modular structure emerged for the mouse mandibles (i.e., the allometric correction diminished the covariation between the hypothesized modules more than the covariation between other subsets of landmarks).

An alternative to the regression approach is the method proposed by Mitteroecker and Bookstein (2007, 2008), which is based on a factor-analytic approach. They identify common factors of variation affecting the whole structure jointly and remove their effect by projection. This method eliminates all the variation in the direction of the shape tangent space that corresponds to the common factors, and therefore removes all variation in that dimension of shape tangent space. A similar projection was also used by Goswami (2006b), who removed the first principal component, which primarily contained variation of size and size-related shape, from size-and-shape data before analyzing integration and modularity.

For external factors that are of a categorical nature (e.g., treatment vs. control, male vs. female, different populations), the correction can be made easily by using the pooled within-group covariance matrix for the analyses of integration and modularity. For computing pooled within-group variances and covariances, the deviations of the observations of the group averages of the variables are used instead of the deviations from the grand mean. Accordingly, this method makes a correction by subtracting the differences among the group means. Versions of this type of adjustment for group difference have been widely used (e.g., Klingenberg et al. 2003; Mitteroecker and Bookstein 2008;). This method assumes that the groups share the same covariance matrix. If this assumption is violated, the pooled within-group covariance matrix may still be a useful compromise between groups, but some caution is advised (e.g., more variable groups have a greater influence on the joint estimate).

### Perspective

Some adjustments to the methodology presented here are necessary for landmark configurations with object symmetry (e.g., Klingenberg et al. 2002), that is, for configurations that are symmetric in themselves. This is often encountered in biological data, for example in studies of skulls. Morphometric data sets of this type consist of paired landmarks on the left and right sides and single landmarks in the midline or median plane (which is also the axis or plane of symmetry). This structure of the data imposes additional restrictions on the way partitions of the landmarks are formed, because the paired landmarks should be included in a subset as pairs, so that corresponding landmarks from the left and right sides either both belong to a subset or are both excluded from it. Similarly, adjacency graphs need to be symmetric, so that corresponding landmarks are either connected or unconnected on both sides. To decide whether a subdivision of landmarks is spatially contiguous, only to the paired landmarks of one side and the unpaired landmarks are considered. For instance, a subset of landmarks from the cheek region can be contiguous even if the mid-facial region that separates the left and right cheeks does not belong to the subset.

The methods introduced in this article are implemented for two- and three-dimensional data in the MorphoJ program package (Klingenberg 2008–2009). The program also incorporates the adjustments for object symmetry.

This article has introduced methods for evaluating hypotheses concerning the boundaries of modules that are given at the outset of the study. It has not considered the problem of an exploratory search for modules in a configuration of landmarks. The same general approach of comparing the strength of covariation among alternative partitions can be used in that context as well, but that application raises a range of additional questions that will be addressed elsewhere.

## References

[b1] Ackermann RR (2005). Ontogenetic integration of the hominoid face. J. Hum. Evol..

[b2] Atchley WR, Cowley DE, Vogl C, McLellan T (1992). Evolutionary divergence, shape change, and genetic correlation structure in the rodent mandible. Syst. Biol..

[b3] Atchley WR, Hall BK (1991). A model for development and evolution of complex morphological structures. Biol. Rev. (Camb.).

[b4] Atchley WR, Plummer AA, Riska B (1985). Genetics of mandible form in the mouse. Genetics.

[b5] Bastir M, Rosas A (2005). Hierarchical nature of morphological integration and modularity in the human posterior face. Am. J. Phys. Anthropol..

[b6] Bastir M, Rosas A (2006). Correlated variation between the lateral basicranium and the face: a geometric morphometric study in different human groups. Arch. Oral Biol..

[b7] Birdsall K, Zimmerman E, Teeter K, Gibson G (2000). Genetic variation for the positioning of wing veins in *Drosophila melanogaster*. Evol. Dev..

[b8] Bolker JA (2000). Modularity in development and why it matters to evo-devo. Am. Zool..

[b9] Bookstein FL (1991). Morphometric Tools for Landmark Data: Geometry and Biology.

[b10] Bookstein FL (1996). Biometrics, biomathematics and the morphometric synthesis. Bull. Math. Biol..

[b11] Bookstein FL, Gunz P, Mitteroecker P, Prossinger H, Schaefer K, Seidler H (2003). Cranial integration in *Homo*: singular warps analysis of the midsagittal plane in ontogeny and evolution. J. Hum. Evol..

[b12] Breuker CJ, Debat V, Klingenberg CP (2006). Functional evo-devo. Trends Ecol. Evol..

[b13] Callebaut W, Rasskin-Gutman D (2005). Modularity: Understanding the Development and Evolution of Natural Complex Systems.

[b14] Cardini A, Elton S (2008). Does the skull carry a phylogenetic signal? Evolution and modularity in the guenons. Biol. J. Linn. Soc..

[b15] Carroll SB, Grenier JK, Weatherbee SD (2001). From DNA to Diversity: Molecular Genetics and the Evolution of Animal Design.

[b16] Cavicchi S, Giorgi G, Natali V, Guerra D (1991). Temperature-related divergence in experimental populations of *Drosophila melanogaster*. III. Fourier and centroid analysis of wing shape and relationship between shape variation and fitness. J. Evol. Biol..

[b17] Chernoff B, Magwene PM, Olson EC, Miller RL (1999). Afterword—Morphological integration: forty years later. Morphological Integration.

[b18] Cheverud JM (1982). Phenotypic, genetic, and environmental morphological integration in the cranium. Evolution.

[b19] Cheverud JM (1995). Morphological integration in the saddle-back tamarin (*Saguinus fuscicollis*) cranium. Am. Nat..

[b20] Cheverud JM (1996). Developmental integration and the evolution of pleiotropy. Am. Zool..

[b21] Cheverud JM, Hartman SE, Richtsmeier JT, Atchley WR (1991). A quantitative genetic analysis of localized morphology in mandibles of inbred mice using finite element scaling. J. Craniofac. Gen. Dev. Biol..

[b22] Cheverud JM, Routman EJ, Irschick DJ (1997). Pleiotropic effects of individual gene loci on mandibular morphology. Evolution.

[b23] Cléroux R, Ducharme GR (1989). Vector correlation for elliptical distributions. Comm. Stat. Theor. Meth..

[b24] Davidson EH (1993). Later embryogenesis: regulatory circuitry in morphogenetic fields. Development.

[b25] Davidson EH (2001). Genomic Regulatory Systems: Development and Evolution.

[b26] de Berg M, van Krefeld M, Overmars M, Schwarzkopf O (2000). Computational Geometry: Algorithms and Applications.

[b27] Dryden IL, Mardia KV (1998). Statistical Shape Analysis.

[b28] Dworkin I, Gibson G (2006). Epidermal growth factor receptor and transforming growth factor-β signaling contributes to variation for wing shape in *Drosophila melanogaster*. Genetics.

[b29] Ehrich TH, Vaughn TT, Koreishi SF, Linsey RB, Pletscher LS, Cheverud JM (2003). Pleiotropic effects on mandibular morphology I. Developmental morphological integration and differential dominance. J. Exp. Zool..

[b30] Escoufier Y (1973). Le traitement des variables vectorielles. Biometrics.

[b31] Gabriel KR, Sokal RR (1969). A new statistical approach to geographic variation analysis. Syst. Zool..

[b32] Gilbert SF, Opitz JM, Raff RA (1996). Resynthesizing evolutionary and developmental biology. Dev. Biol..

[b33] Good P (2000). Permutation Tests: A Practical Guide to Resampling Methods for Testing Hypotheses.

[b34] Goswami A (2006a). Cranial modularity shifts during mammalian evolution. Am. Nat..

[b35] Goswami A (2006b). Morphological integration in the carnivoran skull. Evolution.

[b36] Hooper JW (1959). Simultaneous equations and canonical correlation theory. Econometrica.

[b37] Klingenberg CP (2008). Morphological integration and developmental modularity. Annu. Rev. Ecol. Evol. Syst..

[b38] Klingenberg CP (2008). http://www.flywings.org.uk/MorphoJ_page.htm.

[b39] Klingenberg CP, Badyaev AV, Sowry SM, Beckwith NJ (2001). Inferring developmental modularity from morphological integration: analysis of individual variation and asymmetry in bumblebee wings. Am. Nat..

[b40] Klingenberg CP, Barluenga M, Meyer A (2002). Shape analysis of symmetric structures: quantifying variation among individuals and asymmetry. Evolution.

[b41] Klingenberg CP, Leamy LJ, Cheverud JM (2004). Integration and modularity of quantitative trait locus effects on geometric shape in the mouse mandible. Genetics.

[b42] Klingenberg CP, McIntyre GS (1998). Geometric morphometrics of developmental instability: analyzing patterns of fluctuating asymmetry with Procrustes methods. Evolution.

[b43] Klingenberg CP, Mebus K, Auffray J-C (2003). Developmental integration in a complex morphological structure: how distinct are the modules in the mouse mandible?. Evol. Dev..

[b44] Klingenberg CP, Zaklan SD (2000). Morphological integration between developmental compartments in the *Drosophila* wing. Evolution.

[b45] Kornberg TB, Guha A (2007). Understanding morphogen gradients: a problem of dispersion and containment. Curr. Opin. Genet. Dev..

[b46] Leamy L (1993). Morphological integration of fluctuating asymmetry in the mouse mandible. Genetica.

[b47] Leamy LJ, Routman EJ, Cheverud JM (1999). Quantitative trait loci for early- and late-developing skull characters in mice: a test of the genetic independence model of morphological integration. Am. Nat..

[b48] Lieberman DE, McBratney BM, Krovitz G (2002). The evolution and development of cranial form in *Homo sapiens*. Proc. Natl. Acad. Sci. USA.

[b49] Loy A, Mariani L, Bertelletti M, Tunesi L (1998). Visualizing allometry: geometric morphometrics in the study of shape changes in the early stages of the two-banded sea bream, *Diplodus vulgaris* (Perciformes, Sparidae). J. Morphol..

[b50] Manly BFJ (2007). Randomization, Bootstrap and Monte Carlo Methods in Biology.

[b51] Márquez EJ (2008). A statistical framework for testing modularity in multidimensional data. Evolution.

[b52] Mezey JG, Cheverud JM, Wagner GP (2000). Is the genotype–phenotype map modular? A statistical approach using mouse quantitative trait loci data. Genetics.

[b53] Mitteroecker P, Bookstein FL (2007). The conceptual and statistical relationship between modularity and morphological integration. Syst. Biol..

[b54] Mitteroecker P, Bookstein FL (2008). The evolutionary role of modularity and integration in the hominoid cranium. Evolution.

[b55] Monteiro LR (1999). Multivariate regression models and geometric morphometrics: the search for causal factors in the analysis of shape. Syst. Biol..

[b56] Monteiro LR, Bonato V, dos Reis SF (2005). Evolutionary integration and morphological diversification in complex morphological structures: mandible shape divergence in spiny rats (Rodentia, Echimyidae). Evol. Dev..

[b57] Olson EC, Miller RL (1958). Morphological Integration.

[b58] Palsson A, Gibson G (2004). Association between nucleotide variation in *Egfr* and wing shape in *Drosophila melanogaster*. Genetics.

[b59] Pezzoli MC, Guerra D, Giorgi G, Garoia F, Cavicchi S (1997). Developmental constraints and wing shape variation in natural populations of *Drosophila melanogaster*. Heredity.

[b60] Raff RA (1996). The Shape of Life: Genes, Development and the Evolution of Animal Form.

[b61] Robert P, Cléroux R, Ranger N (1985). Some results on vector correlation. Comput. Stat. Data Anal..

[b62] Robert P, Escoufier Y (1976). A unifying tool for linear multivariate statistical analysis: the *RV*-coefficient. Appl. Stat..

[b63] Rohlf FJ, Corti M (2000). The use of two-block partial least-squares to study covariation in shape. Syst. Biol..

[b64] Schlosser G, Wagner GP (2004). Modularity in Development and Evolution.

[b65] Thompson JN, Woodruff RC (1982). Polygenic analysis of pattern formation: interdependence among veins in the same compartment of the *Drosophila* wing. Genetica.

[b66] von Dassow G, Munro E (1999). Modularity in animal development and evolution: elements of a conceptual framework for EvoDevo. J. Exp. Zool..

[b67] Wagner GP (1996). Homologues, natural kinds and the evolution of modularity. Am. Zool..

[b68] Winther RG (2001). Varieties of modules: kinds, levels, origins, and behaviors. J. Exp. Zool..

[b69] Zelditch ML, Wood AR, Bonett RM, Swiderski DL (2008). Modularity of the rodent mandible: integrating bones, muscles, and teeth. Evol. Dev..

[b70] Zimmerman E, Palsson A, Gibson G (2000). Quantitative trait loci affecting components of wing shape in *Drosophila melanogaster*. Genetics.

